# Supplementary material for: Multiple independent genetic code reassignments of the UAG stop codon in phyllopharyngean ciliates
Source: PLoS Genet. 2024 Dec 17;20(12):e1011512. doi: 10.1371/journal.pgen.1011512 (PMC11687900; doi:10.1371/journal.pgen.1011512)
Supplement: S1 Fig — The table shows log decoding probabilities of UAG for each amino acid. “?” indicates that there were insufficient alignments to infer an amino acid meaning (which is the expected behaviour for stop codons). (PDF) [file pgen.1011512.s001.pdf]

	A	C	D	E	F	G	H	I	K	L	M	N	P	Q	R	S	T	V	W	Y	?
Chilodontopsis depressa	-40.81	-85.74	-30.44	-28.35	-72.81	-56.66	-39.97	-65.48	-33.56	-62.16	-60.60	-27.23	-70.06	-18.73	-46.94	-27.77	-33.36	-63.66	-101.55	-62.09	**0.00**
TARA_ARC_108_MAG_00274	-2091.97	-2351.75	-4215.32	-3690.67	-1488.93	-3845.54	-3169.67	-697.02	-3394.18	**0.00**	-826.24	-3409.71	-3578.15	-2949.29	-3220.11	-2813.36	-2109.52	-979.00	-3050.52	-2468.75	-545.60
TARA_ARC_108_MAG_00306	-3871.08	-4700.99	-7843.32	-6851.27	-2876.66	-7224.96	-5897.50	-1535.69	-6282.83	**0.00**	-1680.20	-6410.23	-6695.74	-5380.27	-6097.99	-5253.27	-3972.26	-2016.61	-5948.80	-4664.07	-952.10
TARA_SOC_28_MAG_00066	-5742.39	-6533.12	-11407.97	-10103.23	-4164.23	-10155.00	-8535.61	-2221.70	-9368.85	**0.00**	-2421.30	-9430.68	-9733.98	-7911.45	-8972.76	-7766.88	-5986.85	-2940.86	-8592.77	-6926.17	-1648.31
Trithigmostoma cucullulus	-25.62	-53.91	-29.53	-22.88	-52.83	-31.17	-26.00	-33.58	-17.40	-40.01	-41.29	-20.16	-45.94	-19.37	-24.64	-17.52	-19.05	-28.76	-60.93	-44.98	**0.00**
Chilodonella uncinata	-27.75	-55.64	-45.10	-30.26	-36.38	-68.98	-32.25	-48.25	-36.52	-40.96	-34.32	-25.47	-58.73	-22.31	-36.99	-22.61	-26.07	-38.80	-55.16	-27.98	**0.00**
Trochilia petrani	-627.48	-1223.45	-699.93	-443.11	-1208.84	-911.80	-572.02	-1158.34	-474.31	-1008.62	-883.22	-516.56	-1097.49	**0.00**	-569.01	-498.23	-584.84	-946.08	-1419.01	-969.87	-264.44
Dysteria derouxi	-27.69	-86.14	-26.97	-7.20	-91.22	-63.46	-18.01	-68.84	-10.89	-59.47	-52.23	-16.74	-57.81	-5.37	-15.83	-18.63	-24.38	-42.87	-93.35	-65.90	**-0.01**
Hartmannula sinica	-2143.62	-4569.62	-2454.62	-1438.33	-4567.37	-3440.24	-2213.33	-4047.66	-1636.42	-3509.28	-3109.71	-1895.60	-3828.68	**0.00**	-1904.59	-1845.04	-2088.89	-3468.06	-5193.11	-3809.87	-933.01
Chilodochona sp	-16.11	-53.56	-42.09	-26.09	-48.72	-42.03	-23.36	-30.42	-14.44	-33.17	-35.05	-19.50	-43.18	-23.25	-25.05	-11.40	-8.59	-22.90	-80.15	-46.11	**0.00**
