# Supplementary material for: Multiple independent genetic code reassignments of the UAG stop codon in phyllopharyngean ciliates
Source: PLoS Genet. 2024 Dec 17;20(12):e1011512. doi: 10.1371/journal.pgen.1011512 (PMC11687900; doi:10.1371/journal.pgen.1011512)
Supplement: S2 Fig — (PDF) [file pgen.1011512.s002.pdf]

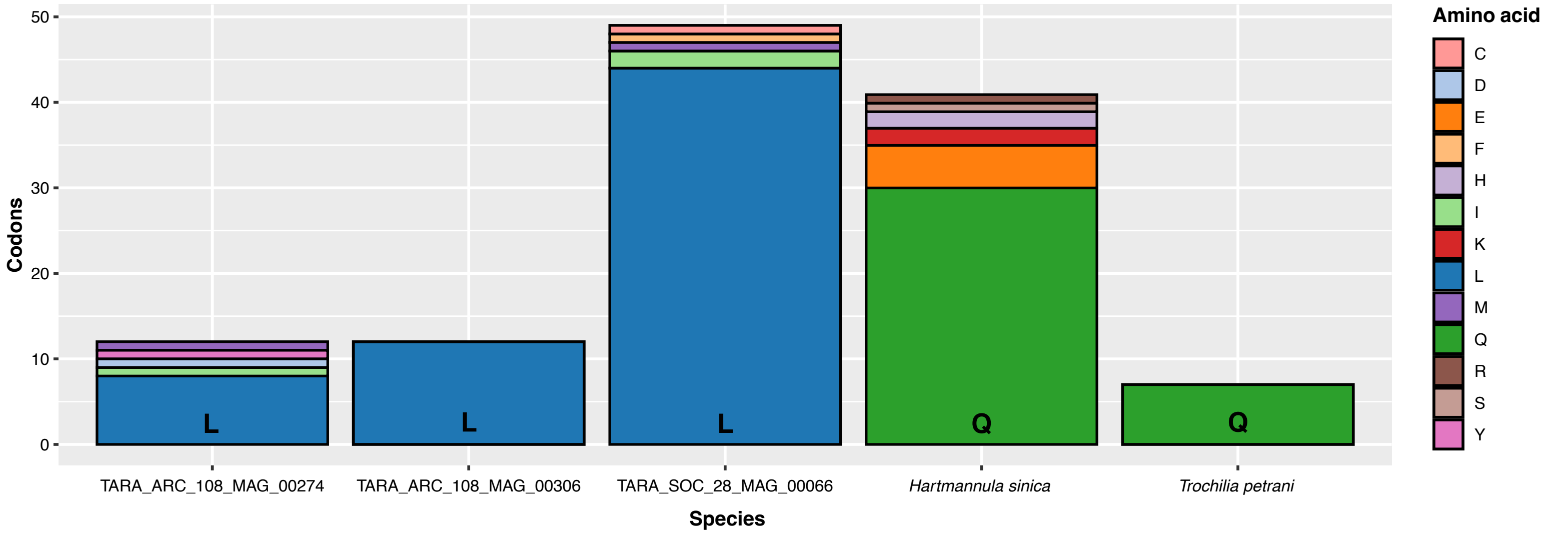

Codons
50
40
30
20
10
0
L
L
L
Q
Q
TARA_ARC_108_MAG_00274
TARA_ARC_108_MAG_00306
TARA_SOC_28_MAG_00066
Hartmannula sinica
Trochilia petrani
Species
Amino acid
C
D
E
F
H
I
K
L
M
Q
R
S
Y
