# Supplementary material for: Multiple independent genetic code reassignments of the UAG stop codon in phyllopharyngean ciliates
Source: PLoS Genet. 2024 Dec 17;20(12):e1011512. doi: 10.1371/journal.pgen.1011512 (PMC11687900; doi:10.1371/journal.pgen.1011512)
Supplement: S3 Fig — (PDF) [file pgen.1011512.s003.pdf]

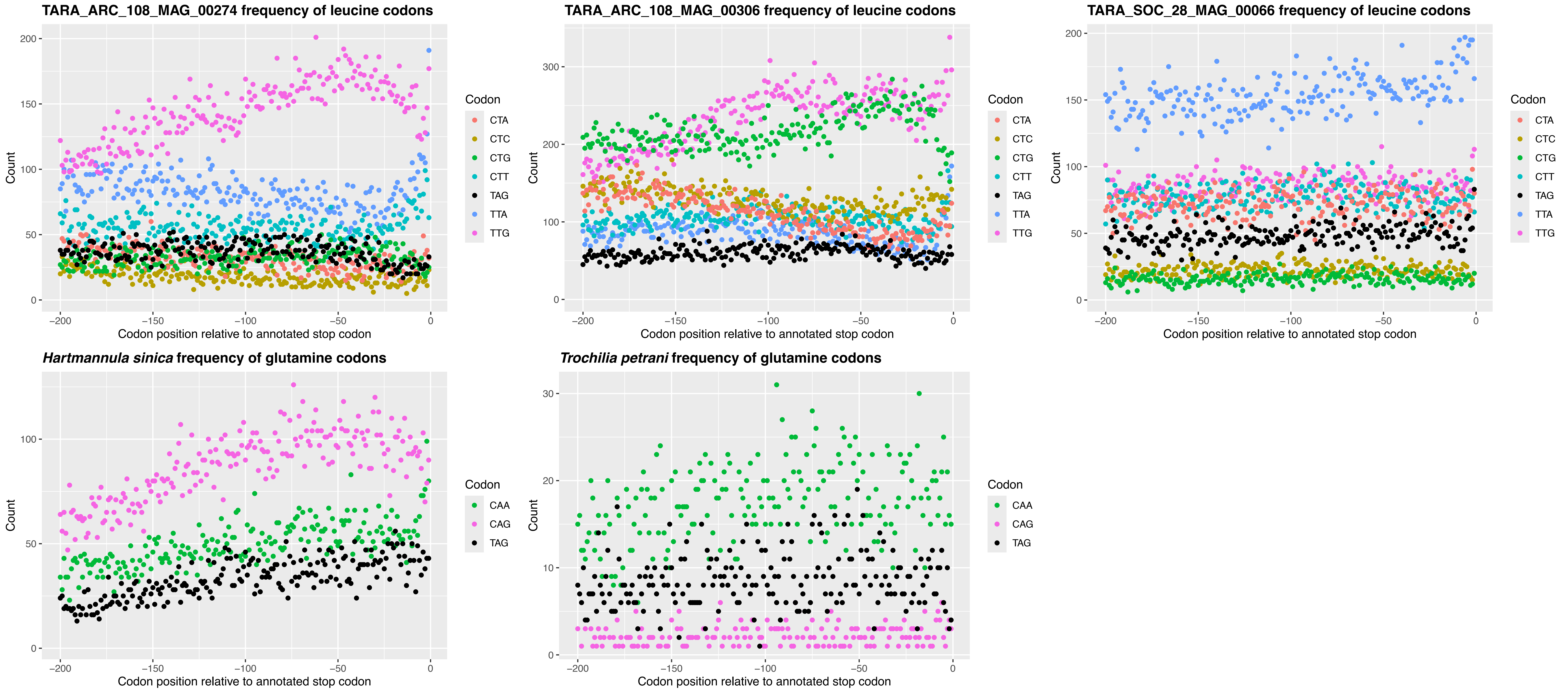
TARA_ARC_108_MAG_00274 frequency of leucine codons
TARA_ARC_108_MAG_00306 frequency of leucine codons
TARA_SOC_28_MAG_00066 frequency of leucine codons
Count
Codon position relative to annotated stop codon
Codon
CTA
CTC
CTG
CTT
TAG
TTA
TTG
Hartmannula sinica frequency of glutamine codons
Trochilia petrani frequency of glutamine codons
CAA
CAG
TAG
−200
−150
−100
−50
0
