# Supplementary figures and images for: Multiple independent genetic code reassignments of the UAG stop codon in phyllopharyngean ciliates
Source: PLoS Genet. 2024 Dec 17;20(12):e1011512. doi: 10.1371/journal.pgen.1011512 (PMC11687900; doi:10.1371/journal.pgen.1011512)

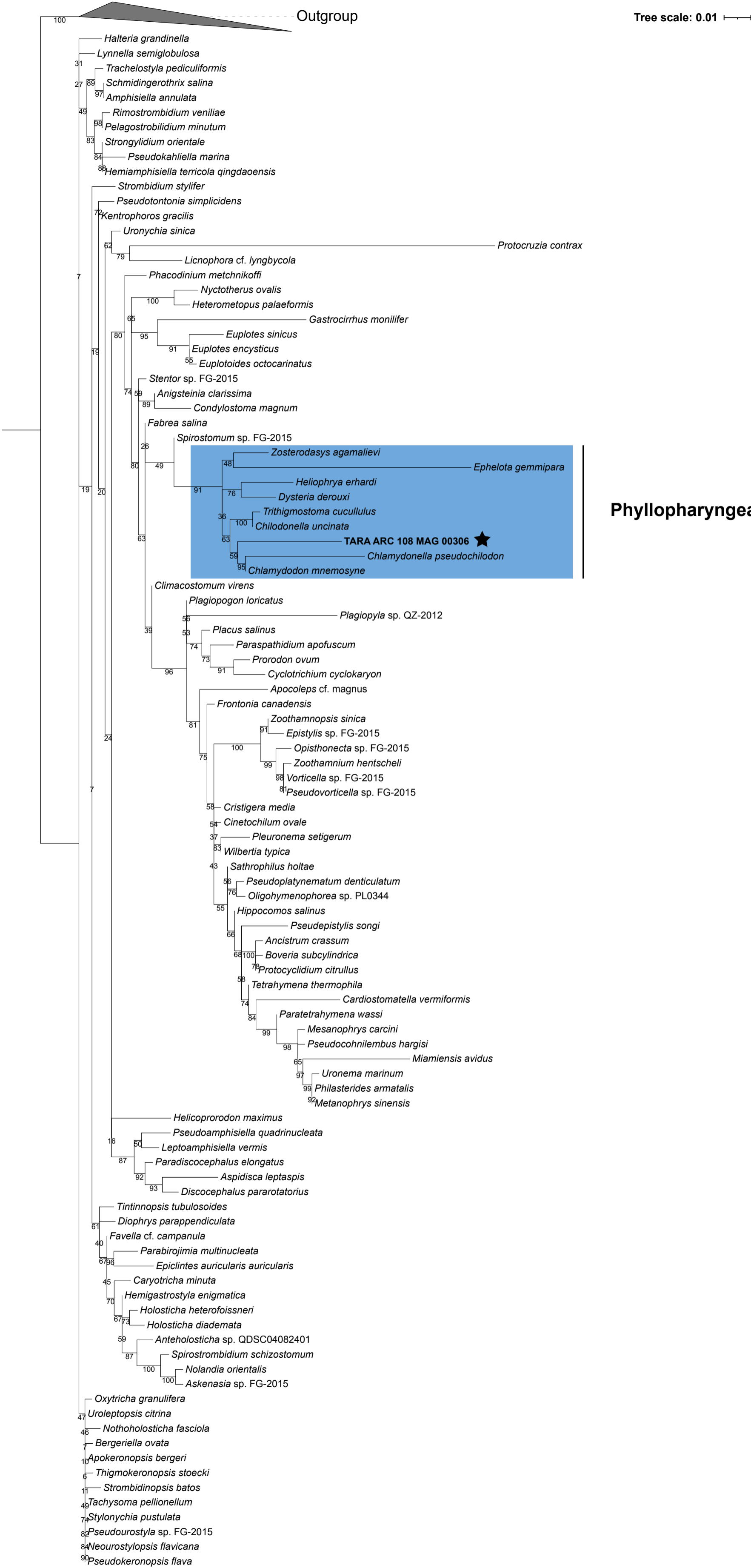

Phyllopharyngea

Supplement: S4 Fig — Numbers represent support from 1000 ultrafast bootstrap replicates. Three apicomplexan sequences were included as an outgroup. (PDF) [file pgen.1011512.s004.pdf]
