# Supplementary material for: Multiple independent genetic code reassignments of the UAG stop codon in phyllopharyngean ciliates
Source: PLoS Genet. 2024 Dec 17;20(12):e1011512. doi: 10.1371/journal.pgen.1011512 (PMC11687900; doi:10.1371/journal.pgen.1011512)
Supplement: S5 Fig — (A) Three suppressor tRNA genes from two TARA Oceans ciliate MAGs (ARC_274 and ARC_306) with CUA anticodons that are predicted to function as leucine tRNAs. (B) Multiple sequence alignment of the three suppressor tRNA genes with representative canonical leucine tRNAs with CAA and TAA anticodons. (PDF) [file pgen.1011512.s005.pdf]

A.

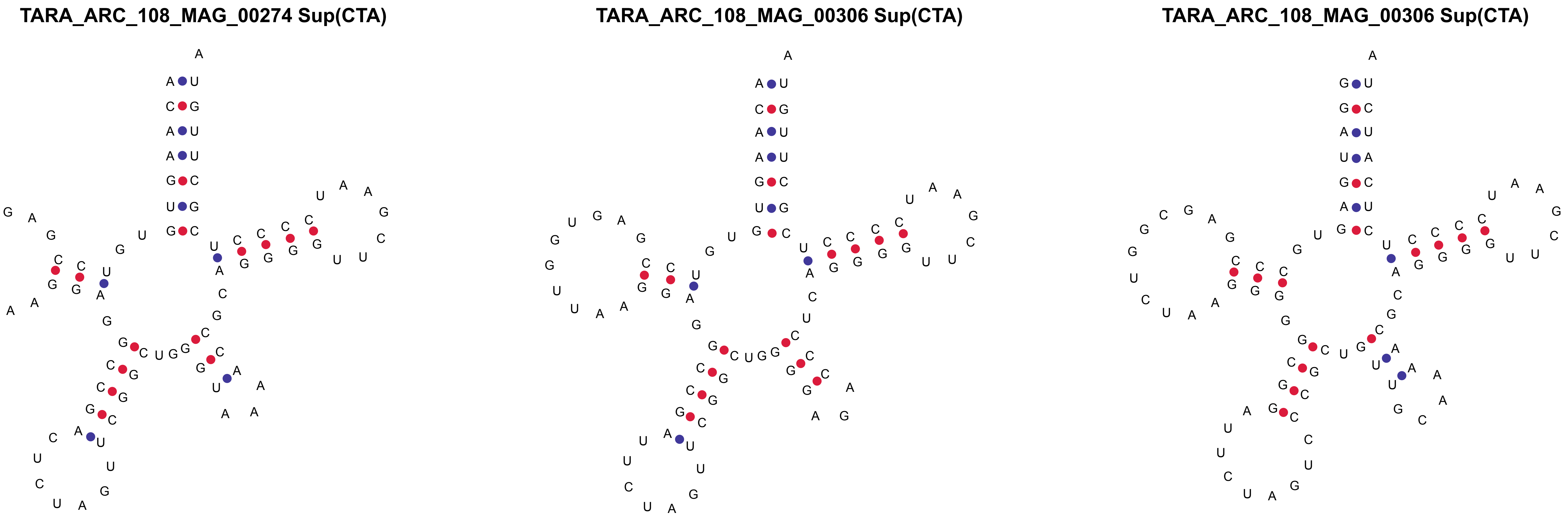
TARA_ARC_108_MAG_00274 Sup(CTA)
TARA_ARC_108_MAG_00306 Sup(CTA)
TARA_ARC_108_MAG_00306 Sup(CTA)

B.

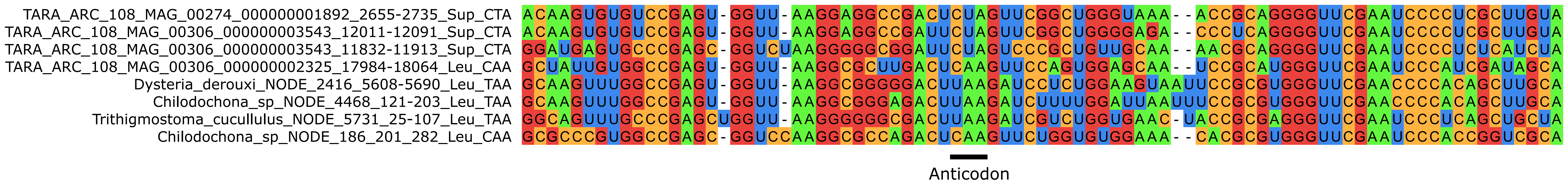
TARA_ARC_108_MAG_00274_000000001892_2655-2735_Sup_CTA ACAAGUGUGUCCGAGU-GGUU-AAGGAGGCCGACUCUAGUUCGGCUGGGUAAA--ACCGCAGGGGUUCGAAUCCCCUCGCUUGUA
TARA_ARC_108_MAG_00306_000000003543_12011-12091_Sup_CTA ACAAGUGUGUCCGAGU-GGUU-AAGGAGGCCGAUUCUAGUUCGGCUGGGGAGA--CCCUCAGGGGUUCGAAUCCCCUCGCUUGUA
TARA_ARC_108_MAG_00306_000000003543_11832-11913_Sup_CTA GGAUGAGUGCCCGAGC-GGUCUAAGGGGGCGGAUUCUAGUCCCGCUGUUGCAA--AACGCAGGGGUUCGAAUCCCCUCUCAUCUA
TARA_ARC_108_MAG_00306_000000002325_17984-18064_Leu_CAA GCUAUUGUGGCCGAGU-GGUU-AAGGCGCUUGACUCAAGUUCCAGUGGAGCAA--UCCGCAUGGGUUCGAAUCCCAUCGAUAGCA
Dysteria_derouxi_NODE_2416_5608-5690_Leu_TAA GCAAGUUUGGCCGAGU-GGUU-AAGGCGGGGGACUUAAGAUCCUCUGGAAGUAAUUCCGCGUGGGUUCGAACCCCACAGCUUGCA
Chilodochona_sp_NODE_4468_121-203_Leu_TAA GCAAGUUUGGCCGAGU-GGUU-AAGGCGGGAGACUUAAGAUCUUUUGGAUUAAUUUCCGCGUGGGUUCGAACCCCACAGCUUGCA
Trithigmostoma_cucullulus_NODE_5731_25-107_Leu_TAA GGCAGUUUGCCCGAGCUGGUU-AAGGGGGGCGACUUAAGAUCGUCUGGUGAAC-UACCGCGAGGGUUCGAAUCCCUCAGCUGCUA
Chilodochona_sp_NODE_186_201_282_Leu_CAA GCGCCCGUGGCCGAGC-GGUCCAAGGCGCCAGACUCAAGUUCUGGUGUGGAAA--CACGCGUGGGUUCGAAUCCCACCGGUCGCA
Anticodon
