# Supplementary material for: Multiple independent genetic code reassignments of the UAG stop codon in phyllopharyngean ciliates
Source: PLoS Genet. 2024 Dec 17;20(12):e1011512. doi: 10.1371/journal.pgen.1011512 (PMC11687900; doi:10.1371/journal.pgen.1011512)
Supplement: Attachment — Submitted filename: McGowan_et_al._2024_review.docx [file pgen.1011512.s009.docx]

Referee’s report on the manuscript “**Multiple Independent Genetic Code Reassignments of the UAG Stop Codon in Phyllopharyngean Ciliates**” submitted to *PloS Genetics* by McGowan et al.

This is a generally well-written manuscript conveying a clear and robustly supported message that three lineages within the ciliate class Phyllopharyngea independently reassigned the codon UAG to encode an amino acid. As such, the study by McGowan is a valuable, albeit not strikingly original, contribution to the literature on the evolution of the genetic code. Indeed, ciliates are known to be extremely prone to change their genetic code in various ways, as also documented by multiple papers from just last two years including a study by McGowan et al. themselves (Chen et al. 2023, doi: 10.1093/molbev/msad064; McGowan et al. 2023, doi: 10.1371/journal.pgen.1010913; Rotterová et al. 2024, doi: 10.1016/j.ympev.2023.107991). Hence, discovering additional such cases is not particularly surprising. Still, I believe the study is worth publishing in *PLoS Genetics*, although I think it first needs to be improved in several directions.

One obvious weakness of the current manuscript is that it does not properly reflect on what has been already known or reported on non-standard genetic code, above all the particular type now reported by the authors from phyllopharyngeans. Most critically, the authors do not mention that Chen et al. (2023; <https://academic.oup.com/mbe/article/40/4/msad064/7084900>) reported the configuration UAG=Q, UAA/UGA=stop, i.e. the genetic code variant identical to *Hartmannula* and *Trochilia*, in the unrelated ciliate *Protocruzia tuzeti* (see Fig. 2 in Chen et al.). Frankly, I have some doubts about some of the results presented by Chen et al., but McGowan et al. cannot really ignore this previously published conclusion and has to properly reflect on it. By this I mean not only that they mention in, but ideally they corroborate or refute the evidence for *P. tuzeti* having the genetic code variant claimed by Chen et al. By doing this they would provide a great service to the community and significantly increase the value of their paper.

Next, for the sake of completeness it is important to mention that Li et al. (2021; <https://academic.oup.com/gbe/article/13/7/evab101/6265471>) described another variant of a nuclear genetic code with in which only UAG is reassigned – in the green algal genus *Scotinospharea*, where UAG encodes serine (in this case the previously published evidence seems to be robust enough). The authors certainly need to mention this and cite the paper, perhaps by inserting extra text into the line 331 before speaking about their own discovery of a ciliate that has reassigned UAG and UAA to two different amino acids.

Additional changes need to be introduced to better present the actual knowledge on the variation of the genetic code in ciliates. Thus, on the line 56 the authors write that UGA is reassigned to tryptophan in *Blepharisma*, but it should be noted that UGA as a Trp codon (as the sole reassigned stop codon) has been reported from several other members of Heterotrichea, such as *Condylostentor auriculatus* (see the paper by Chen et al. cited above). In the following text (line 57) McGowan et al. write that “in … Karyorelictea and *Condylostoma*, all three stop codons have been reassigned”, which is true, but ignores previous results by the authors themselves (McGowan et al., PLoS Genetics 2023) reporting on the same situation (all three stop codons reassigned) in *Plagiopyla frontata* (class Plagiopylea). On the lines 357-358 the authors write “Multiple lineages within Ciliophora have independently evolved the translation of UAR codons to glutamine (McGowan et al. 2023)”, but I think it would be fair to add a citation of a more recent work by Rotterová et al. (2024; <https://www.sciencedirect.com/science/article/pii/S1055790323002919>), which for the first time shows the existence of UAR=Gln reassignment in the ciliate class Armophorea.

In addition, I am not sure if the two tools used for defining the meaning of the UAG codon in phyllopharyngeans can detect situations when the codon has a dual position-dependent meaning as known, e.g., from the ciliate *Condylostoma magnum*. I believe the authors must provide extra evidence that in the three phyllopharyngean lineages with UAG used as a sense codon, it is not at the same time used as a termination codon. Obviously, this cannot be done by simply looking at the coding sequence ends of the gene models McGowan et al. generated by annotating the genome assemblies with a genetic code variant a priori assuming UAG is solely a sense codon. What they have to do is to manually check is assuming UAG solely as a sense codon does not lead to a suspicious extension of the C-termini of the encoded proteins, especially in cases when the C-terminus is highly conserved. It is especially pertinent to carry out such an extra analysis given the claims by Chen et al. (2023) that dual codon meaning is more common in ciliates that thought previously.

Additional issues:

I think it would be wort to mention somewhere that the genetic code variant the authors discovered in *Hartmannula* and *Trochilia* corresponds to what is presented in the NCBI database (<https://www.ncbi.nlm.nih.gov/Taxonomy/Utils/wprintgc.cgi>) and used by many different programmes (such as the Expasy Translate tool, <https://web.expasy.org/translate/>) as the “Blepharisma nuclear” code (or the genetic code 15). Despite the fact that is has been pointed in the literature multiple times hat the early evidence for Blepharisma having UAG as a Gln codon is void, NCBI for unknown reasons still maintains the original totally misleading name of the code variant.

Line 23: change “protistan phyla Ciliophora” to “protistan phylum Ciliophora”

Line 25: I believe you should write “changes in the code”

Line 42: I believe there should be “that” instead of “which” (and if not, I give up my attempts to understand how English works😊)

Line 131: add a dot into “Fig 2”

Line 155: insert “to” into “predicted use”

Line 159: I think the species names “*Hartmannula sinica*” and “*Trochilia petrani*” can be abbreviated to “*H. sinica*” and “*T. petrani*” and referred to like this in the rest of the paper (it’s a common practice to abbreviate species names after they are typed in full at the first mention)

Line 180: “isn’t” – I guess this should be written “is not” in a scientific paper

Line 197: I am not sure about this point, but please check if the article “the” is possibly needed before “sampled Phyllopharyngea”

Line 308: “tRNA loss driven codon reassignment” – I wanted to draw your attention to two problems with this hypothesis: 1) its name is very misleading (as it is also applied to cases where release factor is lost or suffers from a loss-of-function mutation); 2) it is factually identical with the “unassigned codon” model proposed by Sengupta and Higgs in 2005 (see <https://academic.oup.com/genetics/article/170/2/831/6059350>), i.e. more than a decade before Mühlhausen and Kollmar (apparently unaware of the work by Sengupta and Higgs) came with their “tRNA loss driven codon reassignment” model. I think Sengupta and Higgs should receiver proper credit for their work on the evolution of genetics codes.

Line 329: “Rhizarian” – this is a vernacular name and should not be written with the first letter capitalized; the same on line 364

Line 330: “Fornicate” – again, this is a vernacular name and should not be written with the first letter capitalized; the same on line 368

Line 361: do not italicize the citation “(Keeling and Leander 2003)”

Line 362: do not italicize the year “2013”

Line 410: please indicate he version of MAFFT used (if it was always the same, indicate it at this first mention of the programme while you may omit it later)

Line 416: please indicate which “sequence source” (a parameter with multiple options of the search) was used (I guess it was “eukaryotic”, wasn’t it?)

List of references: there are various inconsistencies in the references – journal titles are variously indicated as full names or as abbreviations; in the reference Maurer-Alcalá et al., the correct article details are “*mBio* 9:e01836-17.”; the reference Pan et al. is truncated, the article number (“2819”) is missing after the volume number (see <https://pubmed.ncbi.nlm.nih.gov/31921016/>) – I have seen multiple times that automatic reference editors have the same problem with papers published in Frontiers journals; the correct article details for the reference Sheah et al. are “Peer Community Journal 2:e42”

Figure 3: the name of the taxon “*Oligohymenophora* sp. PL0344” in the tree should be written without italics (“*Oligohymenophora*” is not a genus name) and, for the sake of consistency with the paper by the same authors, the form “Oligohymenophorea” (i.e. with the class-specific suffix “-ea”) should be used.
