# Supplementary material for: Multiple independent genetic code reassignments of the UAG stop codon in phyllopharyngean ciliates
Source: PLoS Genet. 2024 Dec 17;20(12):e1011512. doi: 10.1371/journal.pgen.1011512 (PMC11687900; doi:10.1371/journal.pgen.1011512)
Supplement: Attachment — Submitted filename: Response to Reviewers.docx [file pgen.1011512.s010.docx]

**RESPONSE TO REVIEWERS**

We would like to thank the three reviewers for their thorough reviews of our work. We have incorporated all suggested changes and think they have greatly improved our manuscript and strengthened our findings. Our responses to each reviewer’s comments are detailed below in bold.

**REVIEWER 1**

Reviewer #1: In this paper, McGowan et al report the discovery of novel reassignments of the UAG stop codon in the ciliate class Phyllopharyngea. UAG appears to be reassigned to leucine in a clade of uncultivated ciliates only known from metagenomics, and UAG is additionally reassigned to glutamine in two other Phyllopharyngeal lineages represented by a single species each. These findings add to the already extensive diversity of stop codon reassignments in ciliates, a fascinating system with regards to genetic code evolution. Neither reassignment of UAG to leucine or glutamine in isolation has been previously reported in ciliates. Broadening the diversity of stop codon reassignments known in ciliates is important for future work on why stop codons reassignments are so prevalent in the ciliate clade and on the features that determine codon specificity of eukaryotic release factors.

This claims in this paper are supported by rigorous computational methodology. Since these species are uncultivated, experimental confirmation of these reassignments is out of reach and having multiple lines of computational support is critical. Genetic code predictions were validated by three independent computational approaches using different sets of conserved genes. A consistent suppressor tRNA is found in two of the genomes. Phylogenetic analyses repeated using both ML and Bayesian approaches to establish that the UAG to glutamine reassignments are truly two independent events.

Major comment
- The authors should clarify whether UAG is completely reassigned or whether it may still be used as a stop codon to some degree. These reassignments are currently referred to as if they are unambiguous stop-to-sense reassignments, but no evidence is shown for whether UAG is or isn’t still used as a stop. This is an important point to address in ciliates where dual-sense stop codons have been found in at least two independent lineages. This could be established by an analysis looking at whether UAGs ever occur at positions that align to termination codons in related ciliates. Another analysis that may be interesting to see for the reassigned species is whether UAG usage as a sense codon is depleted in the proximity of the real termination codon (see Fig 5A of Seah et al, 2022 <https://doi.org/10.24072/pcjournal.141> ), which would indicate some remaining context-dependent termination at UAGs. Knowing whether UAG still functions as a stop codon is important for understanding how this reassignment evolved and what mutations to the translational machinery would have been necessary to allow for it.

**Thank you for these suggestions. We have incorporated two additional analyses to address this comment.**

**Firstly, we used an approach that is independent of our genome annotation. We aligned reference proteins from *Tetrahymena thermophila* against each genome assembly and identified alignments where the end of the query protein aligned and checked which codon in the genome assembly that corresponded to. We used different translation tables (1/6/15/16) to assess how that changed our results. With the standard translation table (i.e., treating UAA/UAG/UGA as stop codons), our findings show a clear difference between taxa with the standard genetic code and reassigned codes. In taxa with reassigned UAG codons, most (> ~93%) alignments corresponded to UAA stop codons. Less than 5 alignments per species corresponded to UAG codons, which was comparable to the number that align to other sense codons. When we changed the translation table to 15/16 (i.e., UAG = Q/L) the number of alignments with the end of the query protein aligned increased for taxa with reassigned codes but reduced for taxa with the standard genetic code. We have added text and a supplementary table (Table S2) to describe these results.**

**We also investigated if UAG codons are depleted in proximity of termination codons (which is a hallmark for context dependent codons) and compared the frequency of UAG codons with synonymous leucine/glutamine codons across the gene body. Our results show that UAG is not depleted in proximity with the real termination codon in any of the 5 species with reassigned genetic codes. These findings add further support that it is unlikely that UAG is also used as a stop codon. Furthermore, the frequency evenness of UAG usage across the gene body is similar to that of synonymous leucine/glutamine codons which strengthens our findings that UAG is a sense codon. We have added an additional supplementary figure (Fig. S3) to show these results and have added text to the manuscript.**

**Fig. S3**

Minor comments:
- For the computational genetic code predictions by the various methods, it may be informative to mention the number of codons for the average sense codon and for the other stop codons UAA and UGA, just to establish a reference point for whether the number of in-frame UAGs is similar to other sense codons. Alignment-based approaches could potentially get spurious alignment of stop codons to protein models from recent pseudogenes or sequencing errors.

**We have added a new supplementary table (Table S2) that shows this information from Codetta. It shows the number of codons with Pfam alignments for every codon for each species. We have added text to state that:**

**“The number of UAG codons with an alignment was within the range of other sense codons (Table S2).”**

- In figure 4, dual-sense stop codons are not indicated, making it seem like all of these stop-to-sense reassignments are unambiguous. For example, Plagiopyla frontata, as indicated your previous paper (McGowan et al, 2023), has UGA -> W/*

**We have modified figure 4 to show “UGA 🡪 W/*” for *Plagiopyla frontata*. All other reassignments in our dataset are thought to be unambiguous.**

- In the section about codon usage (lines 225-256), it is unclear how stop codon usage was calculated. For species with predicted UAG reassignments, was UAG considered to be a potential stop codon?

**No, we did not consider UAG to be a potential stop codon for this analysis. During the genome annotation processes we designated UAG as a sense codon as gene prediction tools do not support ambiguous codon meanings (especially in the absence of RNA-Seq data). Furthermore, from our additional analyses above, there is no evidence to suggest that UAG also functions as a stop codon.**

- In lines 216-218, the statement that the suppressor tRNAs are most similar to leucine tRNAs needs to be substantiated more. How is this similarity measured? What other tRNAs were the suppressor tRNAs compared against? How were the tRNA alignments constructed? You can also refer to the literature on tRNA identity to argue that these tRNAs are likely recognized by the leucyl-tRNA synthetase due to the presence of tRNA features (see Giege et al, 2023 <https://doi.org/10.1093/nar/gkad007> ).

**Thank you for this suggestion. In the text we mention:**

**“We compared the suppressor tRNA gene sequences against other phyllopharyngean tRNA sequences in our dataset which revealed that they are most similar in sequence to leucine tRNAs with CAA or TAA anticodons.”**

**We have added text to mention that the suppressor tRNA genes contain many of the known identity elements of eukaryotic leucine tRNAs:**

**“Furthermore, the suppressor tRNA sequences contain many of the known identity elements for eukaryotic leucine tRNAs [23], including the discriminator base A_72_, the positive identity determinants A_4_-U_69_ (not in the second ARC_306 tRNA-Sup which has U_4_-A_69_), G_5_-C_68_, a large variable region, and the invariant nucleotides G_18_, G_19_, A_21_, U_33_, U_54_, U_55_, C_56_, and A_58_ (Fig. S5), suggesting that they are likely to be recognised by leucyl-tRNA synthetases.”**

**Thank you for this suggestion as this strengthens our claim that these novel suppressor tRNAs are likely recognised by leucyl-tRNA synthetases.**

- In Figure S4a, the tRNA secondary structure for the TARA ARC 108 MAG 00306 SupCTA tRNA is incorrect. The variable stem-loop has no loop, which is physically impossible. Instead, the bulged “AA” should be base pairing with the UU, and there should be a GCAA tetraloop at the end. Similarly, the alignment in S4b should be adjusted (especially if arguments about sequence similarity are being based on it). In particular, in the D-loop, the invariant nucleotides G18 and G19 should be aligned to the same column for all sequences. There are designated positions where insertions in tRNAs should be placed (see Sprinzl et al, 1998 <https://doi.org/10.1093/nar/26.1.148> ). A decent alignment can probably be automatically generated by tRNAscan-SE or Infernal. Currently there is no description of how this alignment was generated.

**We have corrected both parts of this figure.**

- In the discussion (lines 292-298), the argument that reverse reassignment back to a stop codon is unlikely is weak. All codon reassignments are deeply unlikely—part of what makes them so interesting is the fact that they do evolve despite being counterintuitive. One could imagine UAG reverting back to being a sense codon, for example, if genomic GC content shifted such that UAG became a very rare sense codon, and now UAG can be reassigned to a stop codon more easily.

**I think this is our point exactly – that reassignments in general are deeply unlikely. It is rare firstly for the UAG codon to be reassigned from stop to sense. Once that change is “fixed” and becomes widely used as a sense codon, switching back to functioning as a stop codon is another unlikely event as it would require genome-wide changes.**

**REVIEWER 2**

Referee’s report on the manuscript “Multiple Independent Genetic Code Reassignments of the UAG Stop Codon in Phyllopharyngean Ciliates” submitted to *PloS Genetics* by McGowan et al.

This is a generally well-written manuscript conveying a clear and robustly supported message that three lineages within the ciliate class Phyllopharyngea independently reassigned the codon UAG to encode an amino acid. As such, the study by McGowan is a valuable, albeit not strikingly original, contribution to the literature on the evolution of the genetic code. Indeed, ciliates are known to be extremely prone to change their genetic code in various ways, as also documented by multiple papers from just last two years including a study by McGowan et al. themselves (Chen et al. 2023, doi: 10.1093/molbev/msad064; McGowan et al. 2023, doi: 10.1371/journal.pgen.1010913; Rotterová et al. 2024, doi: 10.1016/j.ympev.2023.107991). Hence, discovering additional such cases is not particularly surprising. Still, I believe the study is worth publishing in *PLoS Genetics*, although I think it first needs to be improved in several directions.

One obvious weakness of the current manuscript is that it does not properly reflect on what has been already known or reported on non-standard genetic code, above all the particular type now reported by the authors from phyllopharyngeans. Most critically, the authors do not mention that Chen et al. (2023; <https://academic.oup.com/mbe/article/40/4/msad064/7084900>) reported the configuration UAG=Q, UAA/UGA=stop, i.e. the genetic code variant identical to *Hartmannula* and *Trochilia*, in the unrelated ciliate *Protocruzia tuzeti* (see Fig. 2 in Chen et al.). Frankly, I have some doubts about some of the results presented by Chen et al., but McGowan et al. cannot really ignore this previously published conclusion and has to properly reflect on it. By this I mean not only that they mention in, but ideally they corroborate or refute the evidence for *P. tuzeti* having the genetic code variant claimed by Chen et al. By doing this they would provide a great service to the community and significantly increase the value of their paper.

**We have added text to mention that UAG -> Q was reported by Chen et al.**

**“Reassignment of UAG (but not UAA) to glutamine is also reported here to have evolved independently twice within sampled phyllopharyngean ciliates and was also previously reported in the distantly related ciliate Protocruzia tuzeti [7] and the fornicate Iotanema spirale [12].”**

**We have previously looked at the transcriptome data for *P. tuzeti* and decided that there was insufficient evidence at the time to suggest a non-standard genetic code due to the very small number of identified in-frame stop codons occurring at positions that correspond to highly conserved amino acids in other taxa (none in the PhyloFisher eukaryotic dataset for example). However, the custom methods employed by Chen et al. may be more sensitive if this is a rare event. The exact datasets and methods used by Chen et al. are not accessible or easily reproducible. For example, it is not clear if they clustered redundant/alternatively spliced transcripts from their transcriptome assemblies which could inflate the number of apparent genes with detected in-frame UAG codons.**

**We also note that the *P. tuzeti* RNA-Seq dataset used in the Chen et al. paper (SRR7662948 - which comes from an older publication from Sheng et al. 2018;** [**https://www.sciencedirect.com/science/article/pii/S1055790318303348**](https://www.sciencedirect.com/science/article/pii/S1055790318303348)**) has a suspicious number of transcripts (n = 374) that have best hits to *Cafeteria roenbergensis*, suggesting that there may be contamination present. As such, we can neither corroborate or refute the claim by Chen et al. that *P. tuzeti* also uses UAG as a sense codon, until more complete and higher quality data becomes available. Furthermore, the focus on our paper is to explore genetic code evolution within phyllopharyngean ciliates.**

Next, for the sake of completeness it is important to mention that Li et al. (2021; <https://academic.oup.com/gbe/article/13/7/evab101/6265471>) described another variant of a nuclear genetic code with in which only UAG is reassigned – in the green algal genus *Scotinospharea*, where UAG encodes serine (in this case the previously published evidence seems to be robust enough). The authors certainly need to mention this and cite the paper, perhaps by inserting extra text into the line 331 before speaking about their own discovery of a ciliate that has reassigned UAG and UAA to two different amino acids.

**Thank you for highlighting this important reference which we missed. We have now mentioned it in the discussion as suggested and cited it:**

**“Another variant was reported in green algal genus *Scotinosphaera* where UAG encodes serine but UAA is retained as a stop codon [29].”**

Additional changes need to be introduced to better present the actual knowledge on the variation of the genetic code in ciliates. Thus, on the line 56 the authors write that UGA is reassigned to tryptophan in *Blepharisma*, but it should be noted that UGA as a Trp codon (as the sole reassigned stop codon) has been reported from several other members of Heterotrichea, such as *Condylostentor auriculatus* (see the paper by Chen et al. cited above). In the following text (line 57) McGowan et al. write that “in … Karyorelictea and *Condylostoma*, all three stop codons have been reassigned”, which is true, but ignores previous results by the authors themselves (McGowan et al., PLoS Genetics 2023) reporting on the same situation (all three stop codons reassigned) in *Plagiopyla frontata* (class Plagiopylea). On the lines 357-358 the authors write “Multiple lineages within Ciliophora have independently evolved the translation of UAR codons to glutamine (McGowan et al. 2023)”, but I think it would be fair to add a citation of a more recent work by Rotterová et al. (2024; <https://www.sciencedirect.com/science/article/pii/S1055790323002919>), which for the first time shows the existence of UAR=Gln reassignment in the ciliate class Armophorea.

**We have incorporated all these suggested changes to more comprehensively describe the distribution of genetic code variants across other ciliate lineages.**

In addition, I am not sure if the two tools used for defining the meaning of the UAG codon in phyllopharyngeans can detect situations when the codon has a dual position-dependent meaning as known, e.g., from the ciliate *Condylostoma magnum*. I believe the authors must provide extra evidence that in the three phyllopharyngean lineages with UAG used as a sense codon, it is not at the same time used as a termination codon. Obviously, this cannot be done by simply looking at the coding sequence ends of the gene models McGowan et al. generated by annotating the genome assemblies with a genetic code variant a priori assuming UAG is solely a sense codon. What they have to do is to manually check is assuming UAG solely as a sense codon does not lead to a suspicious extension of the C-termini of the encoded proteins, especially in cases when the C-terminus is highly conserved. It is especially pertinent to carry out such an extra analysis given the claims by Chen et al. (2023) that dual codon meaning is more common in ciliates that thought previously.

**Thanks for this suggestion. We have addressed this above in our response to Reviewer 1 showing that there is no evidence to suggest that UAG has a dual position-dependent meaning in phyllophayngeans. We show this by aligning reference proteins against the genome assemblies and determining which stop codon the C-terminus of the reference proteins align to. This approach is independent of our genome annotations. We also show that the frequency of in-frame UAGs is not depleted in proximity with the annotated stop codons (which is a hallmark for codons with dual sense/stop meanings), thus showing that it is unlikely that there is a position/context dependent meaning for UAG.**

**We also added text to the discussion:**

**“In contrast to recent reports that dual context dependent or “bifunctional” stop codons are prevalent in ciliates[7], we found no evidence to suggest that UAG has a dual context dependent meaning in the Phyllopharyngea class of ciliates."**

Additional issues:

I think it would be wort to mention somewhere that the genetic code variant the authors discovered in *Hartmannula* and *Trochilia* corresponds to what is presented in the NCBI database (<https://www.ncbi.nlm.nih.gov/Taxonomy/Utils/wprintgc.cgi>) and used by many different programmes (such as the Expasy Translate tool, <https://web.expasy.org/translate/>) as the “Blepharisma nuclear” code (or the genetic code 15). Despite the fact that is has been pointed in the literature multiple times hat the early evidence for Blepharisma having UAG as a Gln codon is void, NCBI for unknown reasons still maintains the original totally misleading name of the code variant.

**Thank you for this suggestion. We have previously contacted the NCBI to request they correct their genetic code and taxonomy database for ciliates without success. We have added text to the manuscript to mention this point about genetic code 15 (UAG -> Q) and also to mention genetic code 16 (UAG -> L):**

**“This genetic code variant (UAG translated to leucine) is represented by translation table 16 and named the “Chlorophycean Mitochondrial Code” in the NCBI database of genetic codes (**[**https://www.ncbi.nlm.nih.gov/Taxonomy/Utils/wprintgc.cgi)**](https://www.ncbi.nlm.nih.gov/Taxonomy/Utils/wprintgc.cgi))**.”**

**And**

**“This genetic code variant (UAG translated to glutamine) is represented by translation table 15 and named the “Blepharisma Nuclear Code” in the NCBI database of genetic codes (https://www.ncbi.nlm.nih.gov/Taxonomy/Utils/wprintgc.cgi). We note that this name is incorrect and misleading given that Blepharisma do not use this genetic code variant.”**

Line 23: change “protistan phyla Ciliophora” to “protistan phylum Ciliophora”

**Done.**

Line 25: I believe you should write “changes in the code”

**Done.**

Line 42: I believe there should be “that” instead of “which” (and if not, I give up my attempts to understand how English works😊)

**Changed to “that”.**

Line 131: add a dot into “Fig 2”

**Done.**

Line 155: insert “to” into “predicted use”

**Done.**

Line 159: I think the species names “*Hartmannula sinica*” and “*Trochilia petrani*” can be abbreviated to “*H. sinica*” and “*T. petrani*” and referred to like this in the rest of the paper (it’s a common practice to abbreviate species names after they are typed in full at the first mention)

**We initially decided not to abbreviate species names as several taxa in our dataset have similar genus names (e.g., Chilodontopsis, Chilodonella, and Chilodochona) which may confuse readers not familiar with the taxa. However, following the reviewer’s suggestion, and given they are mentioned extensively throughout the paper, we have abbreviated *Hartmannula sinica* and *Trochilia petrani to “H. sinica” and “T. petrani”*.**

Line 180: “isn’t” – I guess this should be written “is not” in a scientific paper

**Done.**

Line 197: I am not sure about this point, but please check if the article “the” is possibly needed before “sampled Phyllopharyngea”

**Added “the”.**

Line 308: “tRNA loss driven codon reassignment” – I wanted to draw your attention to two problems with this hypothesis: 1) its name is very misleading (as it is also applied to cases where release factor is lost or suffers from a loss-of-function mutation); 2) it is factually identical with the “unassigned codon” model proposed by Sengupta and Higgs in 2005 (see <https://academic.oup.com/genetics/article/170/2/831/6059350>), i.e. more than a decade before Mühlhausen and Kollmar (apparently unaware of the work by Sengupta and Higgs) came with their “tRNA loss driven codon reassignment” model. I think Sengupta and Higgs should receiver proper credit for their work on the evolution of genetics codes.

**Thank you for raising this important point. We have added a reference to the paper from Sengupta and Higgs from 2005, and modified the text to reference both papers:**

**“The ‘unassigned codon’ hypothesis [26] and the ‘tRNA loss driven codon reassignment’ [27] hypothesis propose that the genetic code change is preceded by loss of function or reduced efficiency of a tRNA or release factor, resulting in an unassigned or inefficient codon that can be captured by another tRNA gene.”**

Line 329: “Rhizarian” – this is a vernacular name and should not be written with the first letter capitalized; the same on line 364

**Fixed.**

Line 330: “Fornicate” – again, this is a vernacular name and should not be written with the first letter capitalized; the same on line 368

**Fixed.**

Line 361: do not italicize the citation “(Keeling and Leander 2003)”

**Fixed.**

Line 362: do not italicize the year “2013”

**Fixed.**

Line 410: please indicate he version of MAFFT used (if it was always the same, indicate it at this first mention of the programme while you may omit it later)

**We have moved the version info (v7.520) to the first mention of MAFFT.**

Line 416: please indicate which “sequence source” (a parameter with multiple options of the search) was used (I guess it was “eukaryotic”, wasn’t it?)

**Added this information (yes it was eukaryotic search mode).**

List of references: there are various inconsistencies in the references – journal titles are variously indicated as full names or as abbreviations; in the reference Maurer-Alcalá et al., the correct article details are “*mBio* 9:e01836-17.”; the reference Pan et al. is truncated, the article number (“2819”) is missing after the volume number (see <https://pubmed.ncbi.nlm.nih.gov/31921016/>) – I have seen multiple times that automatic reference editors have the same problem with papers published in Frontiers journals; the correct article details for the reference Sheah et al. are “Peer Community Journal 2:e42”

**Thanks for pointing these out. Note that we have updated the referencing style now to match that of PLoS.**

**The article details for our reference to Maurer-Alcalá match that given by the mBio paper - 9:10.1128/mbio.01836-17.
We have fixed the references for Pan et al. and Seah et al.**

Figure 3: the name of the taxon “*Oligohymenophora* sp. PL0344” in the tree should be written without italics (“*Oligohymenophora*” is not a genus name) and, for the sake of consistency with the paper by the same authors, the form “Oligohymenophorea” (i.e. with the class-specific suffix “-ea”) should be used.

**Fixed.**

**REVIEWER 3**

Reviewer #3: In this manuscript McGowan et al have analysed 30 metagenomic assemblies from TARA Oceans project and identified three genomes with UAG codon reassignment decoupled from UAA codon. [They have reported such decoupling in another species two years ago in a publication in the same journal.] Then they assembled additional genomes using raw data obtained from the related species and found additional reassignments. In total three genomes with UAG reassignment to L and two with reassignment to Q. The authors employed two methods for identification of codon reassignments that provide strong support for these findings.

By obtaining sequences from closely related species they were able to reconstruct their phylogenetic relationship and explore the evolution of these reassignments. The analysis suggests that UAG reassignments to Q have occurred independently at least twice. This specific claim relies on the limited genomic data and the accuracy of phylogenetic analysis. I find support for this specific claim relatively weak, but I agree with the authors’ interpretation as the most parsimonious scenario in the light of available data.

The manuscript is sufficiently detailed to enable reproduction of this study and is well written and easy to understand, albeit I have some minor suggestions for improving the text below.

In conclusion, I think it is an important and reliable study that expands our understanding of variant genetic codes diversity in ciliates and its evolution. My comments and suggestions below are intended mostly just to clarify some aspects of this study.

Specific comments:

1. The authors mentioned in Results that they cleaned the data from contaminant sequences before assembly. I couldn’t find the description of how this was done.

**This information is in the methods section under the Dataset assembly section.**

**“Assemblies were decontaminated using a combination of Tiara [41] and contig clustering based on tetranucleotide frequencies.”**

2. The authors report the number of UAG codons corresponding to inferred amino acids in pfam alignments. I still haven’t used Codetta myself and don’t know if it is easy/possible to get information on the number of pfam matches where UAG corresponds to a different amino acid. It would be interesting (of possible) to have Codetta analysis represented in the same way as PhyloFisher (Figure S2).

**According to our knowledge, it is not possible to extract this information from Codetta.**

3. I would appreciate if the authors could provide a bit more detail on selection of specific models for phylogenetic analysis.

**We used IQ-TREE for maximum likelihood phylogenetic analysis. Most sequences (26/29) failed the chi-square test for character composition homogeneity that IQ-TREE runs by default. As such, we used the C20 profile mixture model (LG+C20+F+G) which accounts for different amino-acid compositions along the sequences, thereby better fitting our data's observed compositional heterogeneity. We complemented this approach with a Bayesian phylogeny using PhyloBayes-MPI with the CAT-GTR model, a complex infinite mixture model, to model site heterogenous sequence evolution. Some of the taxa in our dataset have long branches (e.g., *Trichodina pediculus*), therefore the use of such complex models is important to suppress long-branch attraction artifacts. We have added more detail to the text to describe this:**

**"Most sequences (26/29) failed IQ-TREE’s chi-square test for homogeneity of character composition, therefore we carried out maximum-likelihood analysis under the C20 protein mixture model (LG+C20+F+G model) with PMSF approximation [55] and 100 non-parametric bootstraps….”.**

4. The authors discuss the reasons for UAG and UAA coupled meaning in variant genetic codes in the Discussion section and also argue that reassignment of UAA is less likely that of UAG, indeed it has not been observed so far. I fully agree with the authors argument, but I wonder if it will be useful to bring this to introduction to explain why UAG/UAA decoupling is so rare and interesting.

**Thank you for this suggestion. We have expanded our introduction as suggested to highlight why this is so interesting:**

**“In most reported variant genetic codes, the codons UAA and UAG have the same meaning, either they are both reassigned to code for an amino acid or they are both retained as stop codons, suggesting that evolutionary or mechanistic constraints couple the function of these two codons [11,12]. One proposed mechanism for the coupling of UAA and UAG codon meanings is wobble base pairing of suppressor tRNAs. It has been experimentally demonstrated in Tetrahymena thermophila that a suppressor tRNA with a UUA anticodon suppresses both UAA and UAG codons due to wobble base pairing [13]. Thus, if the function of the UAA stop codon was reassigned to function as a sense codon via acquisition of a tRNA-Sup(UUA), it would also likely trigger the reassignment of the UAG stop codon to the same amino acid. Conversely, if UAG were reassigned via acquisition of a tRNA-Sup(CUA), it would not be expected to trigger the reassignment of the UAA stop codon, as the CUA anticodon should specifically recognise UAG [13]. Therefore, genetic code variants where UAA and UAG codons have different meanings are particularly interesting and rare. Such genetic codes require the translational machinery to evolve to efficiently decode one as a sense codon while retaining the other as a termination codon.”**

5. the use of canonical/non-canonical:
“In most reported non-canonical genetic code changes…” such wording may suggest that there are canonical and non-canonical changes of the genetic code. But I am sure that this is not what the authors meant. Overall, if possible, I would advice against using ‘canonical’ and ‘non-canonical’. Besides the theological origin of such language these are vague descriptors. Readers opinions on what is canonical and non-canonical may differ and also change over time. I would suggest using more concrete terms, i.e. ‘standard genetic code’ and ‘variant genetic codes’, the authors do use these terms at places, so it may be also better to stick to them for consistency. I find “Codon usage of canonical and reassigned stop codons” also a bit awkward, one may argue that UAG, UAA and UGA are standard (or canonical if you wish) stop codons and other stop codons (such as AGG in vertebrate mitochondria) are non-standard (non-canonical). But that’s npt what is meant here. I wonder if it will be more accurate to formulate it as something like this “Codon usage of UAA, UAG and UGA specifying stops or reassigned amino acids” or even simpler “UAA, UAG and UGA codons usage”.

**We agree that canonical and non-canonical are perhaps not the best terms to use. We have modified these to “standard” and “variant” throughout the manuscript as suggested.**

**We have also changed the heading/figure legend to “Codon usage of UAA, UAG, and UGA”.**

6. Line 23
“an unusual exception” – to avoid accidental misinterpretation by a uninitiated reader of this being a unique exception, I would consider using a word other than “unusual”, say, something that would emphasize ciliates as being an extreme example of such exceptions for example “the most prominent exception”.

**We have changed this as suggested.**

7. Line 196: Change “that translation of UAG to glutamine” to “that reassignment of UAG to glutamine”

**Done.**

Pasha Baranov
